# Bowel Ischemia in ICU Patients: Diagnostic Value of I-FABP Depends on the Interval to the Triggering Event

**DOI:** 10.1155/2017/2795176

**Published:** 2017-05-28

**Authors:** Stefan Ludewig, Rami Jarbouh, Michael Ardelt, Henning Mothes, Falk Rauchfuß, René Fahrner, Jürgen Zanow, Utz Settmacher

**Affiliations:** Department of General, Visceral and Vascular Surgery, Jena University Hospital, Jena, Germany

## Abstract

**Background:**

Intestinal fatty acid-binding protein (I-FABP) has been shown to be of high diagnostic value in patients with acute mesenteric ischemia. Whether these results can be reproduced in critically ill patients on the ICU was to be investigated.

**Materials and Methods:**

I-FABP was measured in serum and urine of 43 critically ill patients in ICU when mesenteric ischemia was suspected. Bowel ischemia was confirmed in 21 patients (group 1). 22 patients who survived at least seven days without confirmation of ischemia were assigned to group 2. I-FABP levels were compared between the groups, and interval from the event that has triggered ischemia to I-FABP measurement was recorded.

**Results:**

For the identification of patients with mesenteric ischemia, sensitivity, specificity, and area under the curve (AUC) for serum and urine I-FABP were 33.3%, 95.5%, and 0.565 and 81.3%, 70.0%, and 0.694, respectively. I-FABP measurements performed within 12 to 48 h after the event that triggered ischemia showed a sensitivity, specificity, and AUC for serum and urine of 75%, 100%, and 0.853 and 100%, 73.3%, and 0.856, respectively.

**Conclusions:**

In ICU patients, one single I-FABP measurement at the time of clinical suspicion failed to reliably detect or exclude mesenteric ischemia. A higher diagnostic value of I-FABP was only confirmed in the early stages of mesenteric ischemia. I-FABP may be used most appropriately in perioperative monitoring.

## 1. Introduction

Splanchnic hypoperfusion has an important impact on the treatment of critically ill patients in intensive care units, either as transient mesenteric ischemia triggering a gut-derived systemic inflammatory response syndrome (SIRS) or as mesenteric ischemic necrosis [1, 2]. It can be caused by shock situations of various etiologies, for example, major surgery, thromboembolic mesenteric artery occlusion, and burns. Nonocclusive mesenteric ischemia (NOMI) occurs during high-dose administration of vasopressors in intensive care treatment. Since seventy percent of the mesenteric blood flow is directed to the mucosal and submucosal layers of the bowel, these layers are most susceptible to ischemia. The extent of bowel damage may range from mucosal lesions, due to reversible ischemia, to transmural injury, with subsequent necrosis and perforation [3]. The prognosis of mesenteric ischemia is poor with a lethal outcome in up to 60% of patients [4, 5]. One major reason for poor outcome is delay in making the diagnosis due to low specificity of clinical signs and current routine laboratory tests like lactate, pH, base excess, D-dimer, lactate dehydrogenase (LDH), and amylase [6, 7].

Intestinal fatty acid-binding protein (I-FABP) is a valuable marker for epithelial damage of the intestine and, therefore, for mesenteric ischemia. This small cytosolic protein with a molecular weight of approximately 15 kDa is expressed only in mature enterocytes. Due to its low molecular weight and its distribution at the tips of the intestinal villi, it is rapidly released into the circulation and excreted in urine in case of mucosal damage [7]. In humans [8] and rats [9], the highest concentration of I-FABP is found in the small bowel. In the large bowel, the concentration is approximately twenty times lower.

A number of clinical studies demonstrated the high sensitivity and specificity of I-FABP in detecting mesenteric ischemia in patients presenting with acute abdomen [10–12] or in a cardiothoracic surgery perioperative setting [13, 14]. However, the diagnostic value of I-FABP was low in the first study with ICU patients [6].

We conducted a prospective observational trial including critically ill patients in the ICU to address the following questions: Are I-FABP values different in patients with and without mesenteric ischemia at the time of initial clinical suspicion? Does the interval between the event which triggered ischemia and the I-FABP test influence the diagnostic value?

## 2. Material and Methods

### 2.1. Patients

In the multidisciplinary ICU of Jena University Hospital, all patients suspected of having bowel ischemia were included immediately when tested positive for at least one of the following inclusion criteria:

- Clinical signs (prolonged ileus after surgery and increasing need for vasopressors)
- Hyperlactatemia (>3.5 mmol/l or no drop below 5 mmol/l within 12 h after cardiac surgery)
- Signs of bowel ischemia found in endoscopy (ischemic mucosal or transmural necrosis) or computed tomography (pneumatosis intestinalis, occlusion of the superior mesenteric artery, NOMI).

These criteria were compiled from our own clinical experience and have not been restricted to proof of bowel ischemia by means of CT or endoscopy since clinical suspicion often raises due to unspecific signs like delayed bowel function, distension of the abdomen, or hyperlactatemia.

Patients in whom bowel ischemia was confirmed during laparotomy were assigned to group 1 (ischemia). Patients surviving more than 7 days after inclusion into the study without any proof of bowel ischemia were assigned to group 2 (no ischemia). Exclusion criteria were age < 18 years and pregnancy. The study protocol was approved by the local ethics committee of Jena University Hospital (Ref. no. 4028-03/14). Written informed consent was obtained from all patients or their legal representative.

### 2.2. Sample Collection

A urine specimen was taken at the time of study inclusion (specimen “U0”). Blood was collected from the routine blood samples at the following time points: day of inclusion (“d0”), day before inclusion (“d-1”), and the following three days (“d1,” “d2,” and “d3”). No extra blood samples were taken for study purposes. Study samples were aliquoted and stored at −80°C until analysis.

If informed consent could not be obtained within 96 h after study inclusion, all aliquots of the respective patient were discarded.

### 2.3. Laboratory Analyses

Laboratory analyses (serum *n* = 161, urine *n* = 36) were performed after inclusion of the last patient using a commercially available ELISA kit HK406-02 (Hycult Biotech, Uden, Netherlands). The specific measurement of human I-FABP is feasible in the range from 47 to 3000 pg/ml using this assay. Samples > 3000 pg/ml were diluted with buffer solution up to 10 times.

### 2.4. Statistical Analyses

All continuous variables were expressed as median with interquartile range (IQR), since Kolmogorov–Smirnov test showed non-normal distribution of all I-FABP values. The groups were compared by the Mann–Whitney *U* test. Differences in the frequency of patient characteristics were tested for significance with the Chi square test. A *p*  value < 0.05 was considered statistically significant. The cutoff values for the calculation of sensitivity and specificity in the ROC analysis were calculated using the Youden index. The statistical analyses were performed using SPSS Statistics Version 23 (SPSS Inc., Chicago, IL, USA).

## 3. Results

### 3.1. Patients, Diagnoses, and Assignment

During a 9-month period, 2072 patients were treated on ICU. 82 patients met the inclusion criteria. Of 32 patients, informed consent could not be obtained within 96 hours after study inclusion. Six patients were excluded because less than three specimens were available since they died before day 1. One patient without confirmation of mesenteric ischemia died on day 5 and, therefore, could not be assigned to any of the groups. Data of 43 patients were analysed, 21 of them being assigned to group 1 and 22 to group 2. Patients' characteristics and the initial reason for admission into the ICU are listed in Table 1. Groups 1 and 2 included 8 and 15 patients after cardiac surgery, respectively. 30-day mortality in groups 1 and 2 was 52.4% and 22.7%, respectively.

**Figure 1:**
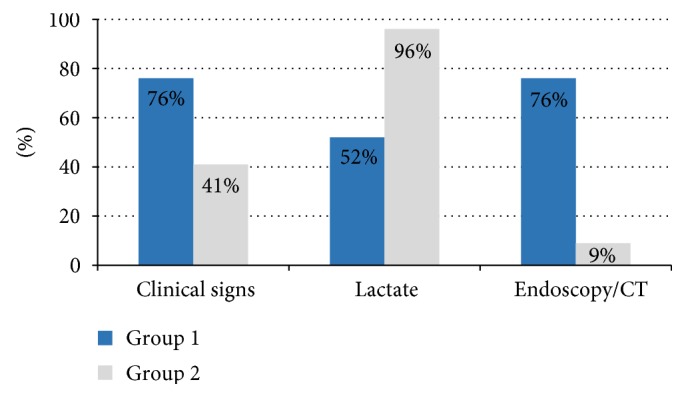
Hyperlactatemia was the most frequent reason for study inclusion in group 2, whereas patients of group 1 frequently had signs of bowel ischemia in endoscopy or CT in addition to clinical suspicion. Only 52% of group 1 had elevated serum lactate levels compared to 96% in group 2.

17 patients met one inclusion criterion (hyperlactatemia only: *n* = 13); 26 patients met two or three inclusion criteria, as shown in Figure 1. 17 patients of group 1 had signs of bowel ischemia in endoscopic and/or CT examinations, but clinical suspicion or hyperlactatemia was evident prior to these in 12/17 patients.

All patients of group 1 underwent laparotomy. In 20 of them, a bowel resection was performed because of ischemia (partial or complete colon resection: *n* = 18, including terminal ileum: *n* = 16, additional small bowel resection: *n* = 6). Mesenteric ischemia was confirmed in all cases by histological examination. Histological findings ranged from ischemia-associated mucosal ulcers to complete bowel necrosis. In one patient with embolic occlusion of the superior mesenteric artery (SMA), ischemia of the small bowel was confirmed by the surgeon and resolved by embolectomy without resection.

### 3.2. Urine I-FABP at Day 0 (U0-Samples)

**Figure 2:**
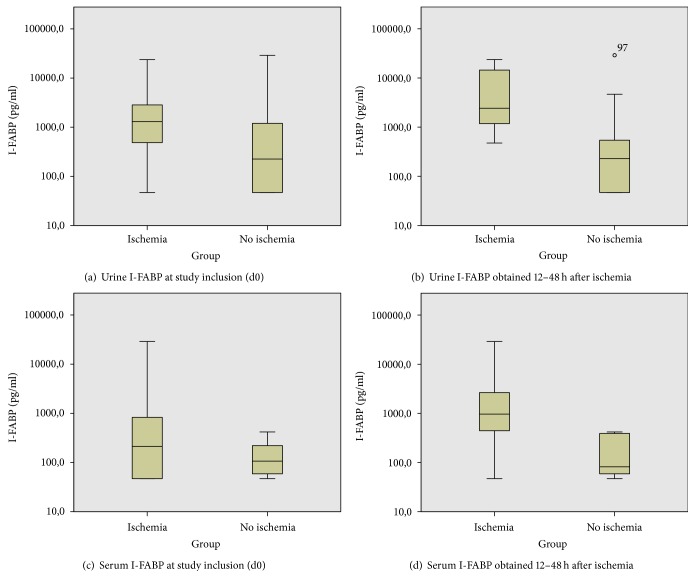
I-FABP at day 0. Comparison of urine and serum samples of both groups. The statistical significance was higher in samples taken 12 to 48 hours after the event that most likely triggered ischemia. Logarithmic scale.

Urine I-FABP was measured in 36 patients. Seven patients had acute or chronic renal failure on the day of inclusion into the study. Median urine I-FABP was significantly different in both groups (1310 pg/ml in group 1 versus 227 pg/ml in group 2, *p* = 0.049, Table 2, Figure 2(a)). One single extreme value > 20,000 pg/ml was observed in each group.

### 3.3. Serum I-FABP at Day 0 (d0-Samples)

In all 43 patients, serum samples of d0 were analysed. Median I-FABP was not significantly different in both groups (213 pg/ml in group 1 versus 109 pg/ml in group 2, *p* = 0.46, Table 2, Figure 2(c)). One single extreme value > 20,000 pg/ml was measured in group 1.

### 3.4. I-FABP Levels and Intervals to Mesenteric Ischemia

In 40 patients, it was possible to identify the event which most probably triggered mesenteric ischemia (major surgery *n* = 33, SMA occlusion *n* = 3, and cardiogenic shock/resuscitation *n* = 4). In the three remaining patients, no potential causative event and no sudden deterioration of the clinical condition within the last 14 days could be identified.

**Figure 3:**
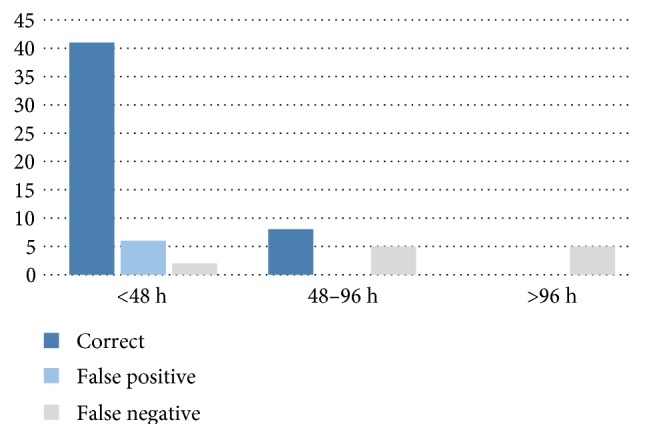
Accuracy of the I-FABP test and its dependence on the interval to the triggering ischemic event, based on all d0 serum and urine samples. Among tests performed later than 48 hours after the event, the number of false negative results increased.

Most I-FABP tests performed within 48 hours after the ischemia-triggering event led to correct or false positive results, as shown in Figure 3. No false positive results were recorded in tests performed later than 48 hours after initial ischemia. However, the percentage of false negative results was increased within this time frame. When the test was performed later than 96 hours after the triggering event, mesenteric ischemia was not reflected by I-FABP test. Based on these findings, we conducted a subgroup analysis using exclusively serum samples (d0) and urine samples (U0) taken within 12 to 48 hours after the event that most likely triggered the mesenteric hypoperfusion. 15 patients were included into the study with an interval of more than 48 hours and 2 patients with less than 12 hours to the triggering event. Results of this subgroup analysis are shown in Table 2 and Figures 2(b) and 2(d).

### 3.5. ROC Analysis, Sensitivity, and Specificity of Serum and Urine I-FABP

Sensitivity, specificity, and AUC were calculated for I-FABP levels at day 0 with analyses of all 43 patients and of the subgroups having their study inclusion 12 to 48 hours after the triggering event.

Table 3 shows the higher diagnostic value of I-FABP tests in the subgroup compared to the total population.

## 4. Discussion

Making the diagnosis of mesenteric ischemia is difficult in ICU patients. Previous studies showed promising results with I-FABP in patients with acute abdomen [10, 11, 15] or abdominal injury [12] and also as monitoring parameter during and shortly after major surgery [13, 14, 16]. We conducted this prospective observational study to evaluate the diagnostic value of I-FABP in ICU patients with suspected mesenteric ischemia.

For the detection of bowel ischemia, urine I-FABP had the highest diagnostic value with a sensitivity and specificity of 81.3 and 70%, respectively. Serum samples of day 0 (study inclusion) showed no statistically significant difference of I-FABP levels in both groups. This may be partially explained by the fact that, according to the study protocol, serum measurements were performed from the daily routine samples which were not always taken at the time of study inclusion.

However, the exact point in time when the mesenteric ischemia occurs is often obscure in ICU patients since clinical signs of acute abdomen are frequently masked. I-FABP is abundant only at the tips of the villi of bowel mucosa and rapidly released into the circulation in case of severe mucosal ischemia [13, 14, 17, 18]. It may be possible that I-FABP is not released when the ischemia of the bowel wall progresses and the mucosa does not recover, leading to false negative I-FABP test results. A similar case can be found in the study of Vermeulen Windsant et al. [13] where one patient with lethal mesenteric ischemia showed normalization of initially elevated I-FABP until day 4 after cardiac surgery. On the other hand, elevation of I-FABP over a short period of time may reflect transient mesenteric hypoperfusion and seems to not necessarily predict development of a transmural bowel necrosis, since regeneration of the bowel is possible when perfusion is restored. In the study of Vermeulen Windsant et al., patients had serum I-FABP levels up to 2300 pg/ml (ELISA kit, Fa. HyCult) during open aortic surgery without developing mesenteric complications. Levels returned to normal until day one after surgery.

The time interval from mesenteric hypoperfusion to I-FABP measurement therefore seems to significantly influence the diagnostic value of the test and may lead to false negative (interval too long) or false positive (interval too short) results. Therefore, one single I-FABP measurement in ICU patients at the time of clinical suspicion failed to reliably detect or exclude mesenteric ischemia in our study. It can be assumed that repeated I-FABP measurements in critical situations like major surgery or cardiogenic shock may better reflect severity of intestinal damage and its recovery. For this clinical applicability, availability of a rapid laboratory assay is needed.

van der Voort et al. evaluated I-FABP test performance in 44 ICU patients with suspected mesenteric ischemia [6]. Medians of 2072 pg/ml in patients developing ischemia and 1020 pg/ml in patients without ischemia (ELISA kit, Fa. HyCult) were not statistically significantly different due to the wide range of I-FABP values. Time intervals from surgery to I-FABP test were not published. High I-FABP levels in both groups were explained with the high prevalence of mesenteric hypoperfusion in critically ill patients.

Symptoms of mesenteric ischemia in ICU patients are often unspecific. Thus, we applied a wide range of inclusion criteria for this study leading to a balanced recruitment to both groups. The retrospective analysis shows that more than 80% of patients of group 1 had signs of ischemia in endoscopy or CT and a large part of group 2 patients was included because of hyperlactatemia. However, in 12 of 17 patients of group 1 who were positive in endoscopy or CT, clinical signs or hyperlactatemia lead to suspicion of mesenteric ischemia and subsequently to further diagnostic measures. In 5 patients, the diagnosis of mesenteric ischemia was based on findings in CT scans that were performed without prior suspicion of mesenteric hypoperfusion. For this reason, we do not think that the unequal distribution of inclusion criteria to both groups influenced the results.

Mesenteric ischemia was confirmed in all patients of group 1 by surgical and histological findings. The definition of the absence of mesenteric ischemia is difficult in general since a comprehensive examination of the entire gut is almost impossible in vivo. Thus, our definition of the negative control group was patient survival of at least 7 days without confirmation of mesenteric ischemia, presuming that a mesenteric ischemia would be fatal within this period. However, some patients of the control group may have had mesenteric hypoperfusion that was resolved and, furthermore, surviving a significant mesenteric ischemia for more than 7 days seems occasionally possible.

The diagnostic value of I-FABP was higher using urine samples as compared to using blood samples. However, critically ill patients in ICU frequently suffer from renal insufficiency impairing both serum I-FABP clearance and urine I-FABP levels. Due to the small heterogeneous sample size, we were not able to investigate the effect of renal function or dialysis on the test results.

Another factor that might influence I-FABP levels to a more or less relevant degree is surgery or manipulation of the bowel. In our study population, 12 patients in both groups had some form of abdominal surgery prior to inclusion to the study (e.g., bowel resection, liver or pancreas resection, and aortic or renal surgery). We did not exclude these patients, because this pilot study aimed to evaluate the performance of the I-FABP test in the real world population of ICU patients. Again, the sample size was too small to perform a subgroup analysis of patients with and without surgical bowel manipulation.

## 5. Conclusions

In ICU patients, one single I-FABP measurement at the time of clinical suspicion failed to reliably detect or exclude mesenteric ischemia. A high diagnostic value of I-FABP was only confirmed in the time interval between 12 and 48 hours after the onset of mesenteric ischemia. I-FABP may be used most appropriately in perioperative monitoring. Further studies should consider the influence of the interval between mesenteric ischemia and I-FABP measurement on the diagnostic accuracy of the test.

## Figures and Tables

**Table 1:** Characteristics of 43 patients at d0 and reason for admission on ICU.

	Group 1 [*n* (%)]	Group 2 [*n* (%)]	*p*
*n*	21	22	—
Male	11 (52.4)	14 (63.6)	0.455
Age [years]	67	65	0.770
Mean BMI	28.37	28.07	0.369
Mean Apache 2 score	22.4	20.6	0.214
On dialysis	10 (47.6)	5 (22.7)	0.087
30 d mortality	**11 (52.4)**	5 (22.7)	**0.044**
Cardiac surgery	8 (38.1)	**15 (68.2)**	**0.048**
Including CPB	4 (19)	8 (36.4)	0.206
Cardiogenic shock	3	1	—
Embolism of SMA	3	0	—
Abdominal surgery	2	1	—
Vascular surgery	2	1	—
Urological surgery	2	1	—
Multiple trauma	1	2	—
Brain surgery	0	1	—

CPB: cardiopulmonary bypass; SMA: superior mesenteric artery; BMI: body mass index.

**Table 2:** I-FABP of serum and urine samples.

	Group	*n*	Median [pg/ml]	IQR [pg/ml]	Minimum [pg/ml]	Maximum [pg/ml]	*p* value
Urine	1	16	1310	2391	47	23,631	**0.049**
	2	20	227	1377	47	29,000	
Subgroup^∗^ urine	1	6	2464	15,781	475	23,631	**0.011**
	2	15	230	848	47	29,000	
Serum day 0	1	21	213	930	47	29,000	0.460
	2	22	109	179	47	420	
Subgroup^∗^ serum day 0	1	8	977	3150	47	29,000	**0.005**
	2	14	82	338	47	420	
Serum day 1	1	21	47	290	47	3294	0.100
	2	22	268	484	47	3998	

^∗^Subgroups contain d0 samples obtained 12 to 48 h after the event that most likely triggered mesenteric ischemia.

**Table 3:** Key figures of the diagnostic value.

	Urine	Subgroup urine	Serum day 0	Subgroup serum day 0
Sensitivity	81.3%	100%	33.3	75%
Specificity	70%	73.3%	95.5	100%
AUC	0.694	0.856	0.565	0.853
Cutoff [pg/ml]	402.2	402.2	410.3	410.3

## Abbreviations

AUC: Area under the curve
BMI: Body mass index
CPB: Cardiopulmonary bypass
CPR: Cardiopulmonary resuscitation
CT: Computed tomography
ELISA: Enzyme-linked immunosorbent assay
ICU: Intensive care unit
I-FABP: Intestinal fatty acid-binding protein
LDH: Lactate dehydrogenase
NOMI: Nonocclusive mesenteric ischemia
SIRS: Septic inflammatory response syndrome
SMA: Superior mesenteric artery
TAVI: Transcatheter aortic valve implantation.
